# Mobile phone applications to overcome malnutrition among preschoolers: a systematic review

**DOI:** 10.1186/s12911-019-0803-2

**Published:** 2019-04-05

**Authors:** Navisa Seyyedi, Bahlol Rahimi, Hamid Reza Farrokh Eslamlou, Toomas Timpka, Hadi Lotfnezhad Afshar

**Affiliations:** 1 Student Research Committee, Urmia University of Medical Sciences, Urmia, Iran; 2 Department of Health Information Technology, Urmia University of Medical Sciences, Nazloo Campus, Sero Road, Urmia, Iran; 3 Department of Public Health, Urmia University of Medical sciences, Urmia, Iran; 4 Department of Computer and Information Sciences, Linköping University, Linkoping, Sweden; 5 Department of Medical and Health Sciences, Linköping University, Linkoping, Sweden

**Keywords:** Mobile phone, Malnutrition, Intervention, Preschoolers

## Abstract

**Background:**

Malnutrition is one of the most important reasons for child mortality in developing countries, especially during the first 5 years of life. We set out to systematically review evaluations of interventions that use mobile phone applications to overcome malnutrition among preschoolers.

**Methods:**

The review was conducted and reported according to the Preferred Reporting Items for Systematic reviews and Meta-Analyses: the PRISMA statement. To be eligible, the study had to have evaluated mobile phone interventions to increase nutrition knowledge or enhance behavior related to nutrition in order to cope with malnutrition (under nutrition or overweight) in preschoolers. Articles addressing other research topics, older children or adults, review papers, theoretical and conceptual articles, editorials, and letters were excluded. The PubMed, Web of Science and Scopus databases covering both medical and technical literature were searched for studies addressing preschoolers’ malnutrition using mobile technology.

**Results:**

Seven articles were identified that fulfilled the review criteria. The studies reported in the main positive signals concerning the acceptance of mobile phone based nutritional interventions addressing preschoolers. Important infrastructural and technical limitations to implement mHealth in low and middle income countries (LMICs) were also communicated, ranging from low network capacity and low access to mobile phones to specific technical barriers. Only one study was identified evaluating primary anthropometric outcomes.

**Conclusions:**

The review findings indicated a need for more controlled evaluations using anthropometric primary endpoints and put relevance to the suggestion that cooperation between government organizations, academia, and industry is necessary to provide sufficient infrastructure support for mHealth use against malnutrition in LMICs.

## Background

Malnutrition is one of the most important reasons for child mortality in developing countries, especially during the first 5 years of life. Malnutrition is a condition of deficiency or excess of nutrients which leads to sensible adverse effects on body composition and functionality [1–3]. According to World Health Organization (WHO) reports, 52 million children under 5 years of age are wasted, 17 million are severely wasted and 155 million are stunted, while 41 million are overweight or obese [4]. It is well known that early deficiencies in the intake of nutrients not only have a negative influence on child health and development, but it also reduces work efficiency and leads to poor reproductive outcomes in adult life through impaired immune response and susceptibility to infections, digestive problems, predisposing for vicious cycles of recurring sickness, faltering growth and diminished learning ability [5–8]. On the other hand, children who are overweight during their pre-school years tend to remain overweight throughout childhood and adolescence, which increases the risk suffering of cardiovascular disease, several forms of cancer, diabetes, and other chronic illnesses [9, 10]. The causal chain leading to malnutrition in early childhood is complex with a variety of direct and underlying contributors including household access to food and the distribution of this food within the household, inadequate maternal and child care practices, and poor access to health-care services [11, 12]. Knowledge of these factors is an essential prerequisite for determining intervention programs addressing preschoolers malnutrition [13]. A recent review of factors influencing information and communication technology (ICT) introduction in health services showed that one main type of technology that has been adopted by broad categories of healthcare users and patients is mobile and cell phone technologies and these kind of studies are going to be published broadly [14, 15]. At present, mobile technologies allow development of mHealth (mobile health) interventions that have promised to be a useful and low-cost way to disseminate information about proper nutrition and a critical source of motivation for behavioral change [16, 17]. A systematic review has investigated the effect sizes of pediatric obesity intervention studies using of mobile technology within the elementary school students. Based on four included studies, analyses showed that the mobile interventions positively affected dropout rates, but had no influence on weight control, exercise, and sugar-sweetened beverage intake [18]. Another systematic review of the effectiveness of using smartphones on child and adolescent overweight reported a significant effect on Body Mass Index (BMI) z-scores [19]. Furthermore, a recent review identified a number of weight management applications developed for children > 12 years. Most were free or inexpensive to download and accordingly attractive for parents of obese children. The investigations found the overall quality of the application contents generally being very poor and not based on credible dietary guideline [20]. To our knowledge, no reviews have been conducted on dietary interventions based on mobile phone applications addressing preschoolers’ malnutrition.

We set out to systematically review evaluations of interventions that use mobile phone applications to overcome malnutrition among preschoolers.

## Methods

The review was conducted and reported according to the Preferred Reporting Items for Systematic reviews and Meta-Analyses: the PRISMA statement [21].

### Inclusion and exclusion criteria

To be eligible in this review, the study had to have evaluated mobile phone interventions to increase nutrition knowledge or enhance behavior related to nutrition in order to cope with malnutrition (under nutrition or overweight) in preschoolers. Articles addressing other research topics, older children or adults, review papers, theoretical and conceptual articles, editorials, and letters were excluded.

### Search strategy

A literature search procedure was carried out in March 2018 based upon bibliographic searches in the PubMed, Web of Science and Scopus databases covering both medical and technical literature. English-only articles without any date restriction were identified using the following MeSH terms: (“Child Nutrition Disorders” OR Malnutrition OR “child Weight” OR “Pediatric Thinness” OR “Pediatric Obesity” OR “Pediatric Feeding Disorders”) AND (“Mobile Applications” OR smartphone OR “cell phones” OR “mobile phones” OR mobile OR “portable software app”). Additionally the following key journals in the health informatics sciences (Journal of the American Medical Informatics Association, International Journal of Medical Informatics, Journal of Medical Internet Research, and Telemedicine and e-Health) were manually searched to prevent the likelihood of omitting relevant articles. The author, title, journal, year of publication and abstract for each article were compiled into a single EXCEL file. By removing duplicates only 1775 articles remained.

**Fig. 1:**
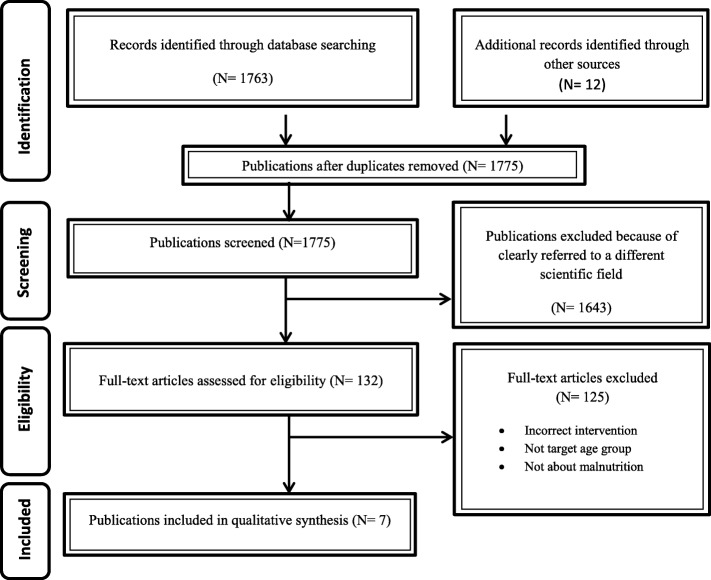
Flow diagram of study selection

Our initial search identified 296 titles from PubMed, 667 from Web of Science and 800 from Scopus; of these, only seven articles met all search criteria (Fig. 1). The references of the included studies were retained in a list to further reduce the likelihood of missing relevant articles. Finally, data were extracted and evaluated (design, outcomes, sample size, duration of intervention and advantages of and barriers to using mobile devices) by three independent researchers. The results were organized into four main emerging themes for clarity of presentation.

## Results

The seven articles fulfilling the review criteria reported studies with designs ranging from case studies to RCTs. All articles were published between 2015 and 2017. Four of the reported interventions were conducted using a mobile phone application, and two interventions used the combination of SMS on mobile phone and an ICT tool or face-to-face visit interventions. Table 1 shows the main characteristics of the included publications.

**Table 1:** Selected publications

Authors & (Publication Date)	Study design	Study objective	Intervention	Intervention setting & Population	Main study results & endpoints	Weaknesses and limitations	Strengths
Hull et al. [22]. 2017 Aug	Prototype, Usability testing	To test the application prototype with target users, focusing on usage, usability, and perceived barriers and benefits of the app.	A prototype designed application that includes some shopping tools such as a barcode scanner and calculator tools for the cash value voucher for purchasing fruits and vegetables, and nutrition education focused on healthy snacks and beverages	(African Americans and Hispanics in the United States) (80 mothers of children ages 2 to 4.5 years) (3 months duration)	The app prototype successfully demonstrated the feasibility of using it. Although, Some technical barriers reported by users. Also a few of them indicated more problems with the app not being easy enough to use, lack of interest in the content, forgetting to use it, or not noticing alerts.	1- Compatibility issues with different Android platforms 2- Limited support of vendors 3- technical barriers and delay in updating the database	1- High willingness to shopping tools including a barcode scanner and calculator tools for the cash value voucher for purchasing fruits and vegetables 2- improving food purchasing behaviors, parent feeding strategies
Nyström et al. [23]. 2017 April	Randomized Controlled Trial (RCT)	To assess the effectiveness of a mHealth obesity prevention program on body fat, dietary habits, and physical activity in healthy Swedish children aged 4.5 y.	The application consisted of an extensive program of information and support grounded in social cognitive theory and behavior change techniques and was centered around existing guidelines for healthy eating and physical activity	(Ostergotland, Sweden) (315 preschool-aged children were randomly assigned to an intervention or control group.) (Parents in the intervention group received a 6-mo mHealth program.)	No statistically significant intervention effect was observed for Fat Mass Index (FMI) between the intervention and control group. However, the intervention group increased their mean composite score from baseline to follow-up, whereas the control group did not.	1- Children of normal weight were included, which may have diluted the effect of the intervention on FMI. 2- the intervention was provided only in Swedish	1- compatible with both iOS and Android operating systems 2- intervention was grounded in social cognitive theory 3- careful calculation of statistical power 4- validated measures for dietary intake
Shah et al. [24]. 2016 Dec	Pilot study with quantitative approach	To test the feasibility and acceptability of such ICT based approach.	Mobile based videos for training of all the (health) field workers to increase the nutritional and health status of children between 0 and 6 yrs.	Jogmodi beat in India was selected for the pilot. Jogmodi beat had 25 AWWs (Anganwadi Workers). (4 weeks duration)	The feedbacks indicated that old unhealthy practices are still followed in the village in the name of religion and old traditions. According to reports the major barrier was availability of communication network problem in their village.	1- low availability of communication network 2- The role of the supervisor was not clear that needs to be planned in the way forward	1- Using videos as an effective media 2- Intervention developed in various local languages which cover all topics relating to malnutrition and its care. 3- Giving recharge for calling for the oral exam
Weerasinghe et al. [25]. May 2016	Formative study with qualitative approach	To understand the nature of mobile phone use and perceptions of m-health for infant and young child feeding counseling among the mothers, their family members, and service providers	using m-health counseling for infant and young child feeding	(Sri Lankan Tea Estates) (27 focus group discussions with mothers, fathers and grandmothers of the children under the age of 5) (15 in-depth interviews with health care team)	Mothers’ perception were positive about receiving health related massages and reminders on Child Feeding through phones. On the other hand, Health workers were willing to mobile intervention as a supplementary method to face-to-face interaction. According to results m-health platform could be a promising initiative to improve the nutritional status of children.	1- Low availability of mobile phones 2- Distribution of signal strength is not adequate in some area	1- Mothers’ tendency to receive mobile-technology based counseling. 2- health workers were willing to use mobile phones as a complementary to strengthen communications
Militello et al. [26]. (October 1, 2015)	Case study (mostly qualitative approach)	1. To assess correlations among the study variables (healthy lifestyle beliefs, perceived difficulty, and healthy lifestyle behaviors) in parents of overweight /obese preschool children. 2. If the parent’s level of cognitive beliefs and perceived difficulty of engaging in healthy lifestyle behaviors correlated with text messaging cognitive behavioral support.	The intervention relies on Cognitive Behavioral Skills Building (CBSB) to include nutrition and physical activity knowledge, problem solving, goal setting, effective communication, positive self-talk, and positive thinking. The program was delivered through a combination of clinic visits, homework/ reinforcement, reminders (manual or audio option), and text message triggers.	(Columbus, USA) Sample of 15 parent-preschooler dyads (13 parents and 15 overweight/ obesity preschoolers aged 3 through 5 years)	This finding indicated that the parents’ level of cognitive beliefs and perceived difficulty of engaging in healthy lifestyle behaviors correlated with the text messaging cognitive behavioral support. As parental healthy lifestyle beliefs increased and perceived difficulty lessened, their response rate and subsequent feedback lessened to the static text messaging support.	1- using text messaging (SMS) that considered to be not effective as a mobile application because of its constraints 2- It is unknown whether the SMS were perceived by participants as motivators, facilitators, or reminders to act.	1- Utilizing Beck’s Cognitive Theory for intervention content 2- Utilizing Fogg’s Behavior Model for the implementation.
Denney-Wilson et al. [27]. BMJ open. 2015 Nov 1	Non-randomized quasi experimental	To assess: 1. The feasibility of PHC practitioners referring parents to and incorporating an m-health intervention and reinforcing key messages as part of routine baby health checks; 2. The effectiveness of an m-health intervention in terms of its reach, use, acceptability, cost and impact on key infant nutrition and feeding outcomes.	The program is a smartphone app, website and online forum providing parents living in socioeconomically disadvantaged areas with a ‘one-stop shop’ for evidence based advice and tips, consistent with national guidelines on infant feeding in the first 9 months of life.	(200 parent/child dyads to the intervention arm) (infant age less than 3 months, 6 months and 9 months)	Child Health nurses’ staff acknowledged that the content was consistent with guidelines and agreed to participate in the study and refer parents to the app.	1- Because of study budget and timeframe limitations, intervention term is restricted to 9 months of age that is not sufficient to get desirable effects	1- Developing the intervention content tailored to age of child. 2- Using sound theoretical framework. 3- the content consistency with guidelines 4- Utilizing recruitment strategies to Motivate participants into the intervention
Charles et al. [28]. 2016 IEEE/ACIS 15th International Conference	Case study with qualitative approach	The study uses both theoretical and practical approach to define the factors that fuel malnutrition and its other cause, how Information and Communication Technology tools can help to grasp the fact. Therefore, the developed tool will mainly have an SMS platform.	The Online Nutrition Surveillance System gives a possible push and pull system where information is gathered and retrieved upon request by either SMS or the web-based portal and visiting the nearest tele-center as a means of both advocacy and information sharing to minimize the information gap consequently improve household knowledge .	50 respondents were selected from the major sub-counties of western Uganda to facilitate the research process about a population of concern.	Regarding the study, SMS platform, Discussion forum, Mailing list and more components of the web-based portal were suggested as effective ways to expand awareness and knowledge sharing about malnutrition.	1- Limited capabilities due to weakness in available ICT infrastructure. 2- Enormous challenges like lack of a database and information system, inadequate standardized data collection	1- focus on finding the most appropriate ways of information distribution of malnutrition best practices 2- Utilizing the combination advantages of SMS platform, Discussion forum and Mailing list. 3- Using SMS on mobile phone as an alternative for individuals that can’t afford a smart phone to access a web-portal

### Participants’ perceptions of mobile phone use for nutritional counseling

The study participants that provided their perceptions of issues and attitudes related to preschoolers feeding and use of mobile phones were mainly mothers and healthcare workers.

#### Mothers’ perceptions of nutritional counseling by mobile phone

A study performed in a poor region of Sri Lanka reported that only a small proportion of mothers to preschoolers owned personal mobile phones or had access to their husband’s mobile phone. Signal strength was different in various parts of the region and in some areas it was very low. According to the women’s perception, they were assured that nobody would disagree if they used a mobile phone for purposes such as contacting the husband and Public Health Midwives (PHM) or getting health information. They stated that they used stationary phones, and had not experienced any need to acquire a mobile phone. The mothers were agree about receiving health-related massages and reminders on Infant and Young Child Feeding (IYCF) by phone and they were willing to learn the new technology for the benefit of their children [22]. Also from another study, the mothers’ interventional behaviours were reported to be affected by their opportunities, capabilities, and motivation. They tended to receive positive, affirming and personalized messages [23]. This pattern was also reflected in the Mobile-based Intervention Intended to Stop Obesity in Preschoolers (MINISTOP) RCT, where the mothers’ participation appetency was slightly lower in the youngest age group and also low-income families [24].

#### Health workers’ perceptions of nutritional counseling by mobile phone use

Health workers in a poor region of developing country (Sri Lanka) have been reported to perceive that mobile messaging, voice or text, mainly should be a supplementary method to face-to-face interaction. They were not sure about their capability of using the technology for complex tasks, such as entering data, if required in a mHealth initiative [22]. In comparison, in the work-up for the Growing healthy study, Child health nurses were found to be willing to learn from feedback provided by the application. They also acknowledged that the content was consistent with concurrent guidelines and agreed to refer parents to the application [23].

### Technical and usability feedbacks

Technical issues were reported by mothers to make a specific feature or an entire mobile phone barcode scanner application impossible to use. These issues were minor phone damage, installation failure, and features with incorrect operation (*bugs*). A few mothers indicated that dissatisfaction with the applications not being user friendly also lessened their interest in the educational content and alerts. However, the major problem with the application was identified as barcode scanner incompatibility with Android phones. Some errors and updating delay were also reported in database of WIC-approved (Women, Infants, and Children) items identified by the grocery store chains. Moreover, the Yummy Snack Gallery (designed to provide parents practical skills to improve healthy dietary for children) gained high scores on the measures such as ease of use, supportiveness, efficiency, and satisfaction [25]. However, the collected feedback results showed that several participants could not continue the program because of low communication network availability in their region. The other barriers to continued participation included, for example, video deleted by error and inaccessibility to videos in spouse’s mobile phone working out of town [26].

### Factors affecting m-health intervention usage and adoption

A case study aimed to investigate associations between mothers’ healthy lifestyle beliefs, perceived difficulty, and healthy lifestyle behaviors with regards to their overweight preschoolers. The results showed reverse relationship between healthy lifestyle beliefs and perceived difficulty to apply those behaviors. The study continually assessed the correlation between lifestyle variables and cognitive behavioral support through text messaging. Stronger beliefs correlated with a lower tendency to respond to the static SMS and automated feedback. Similarly, less perceived difficulty was related to lower reaction rate to the text messaging [27].

ICT systems acceptance is known to be associated with challenges concerning technological and attitude aspects [15]. In a large-scale requirements analysis addressing information systems providing decision support with regards to malnutrition issues, 50 respondents were selected from the major sub-countries of western Uganda. The study revealed significant weaknesses in available ICT infrastructure and major challenges associated with lack of a database technology and standardized means for data collection [28].

### Efficacy (impact) of m-health intervention on child weight

Only one study (MINISTOP) reported efficacy outcomes from a controlled evaluation. The primary outcome was Fat Mass Index (FMI). While the secondary outcome was a combination of scores such as consumed fruits, vegetables, candy, and sweetened beverages as well as physical activity time span. However, the primary outcome between the intervention and control groups had no change but the intervention group could improve the composite score ultimately. The children with a higher FMI had been affected by the intervention more than others because these children need it more [24].

## Discussion

The review revealed from evaluations of mobile phone based interventions to improve nutritional conditions among preschoolers that although mobile communication is now part of the everyday life in most global settings, the use of mHealth applications to provide health information and care is still challenging. It has been pointed out that the essential considerations for successful implementation of mHealth innovations include consideration of local infrastructure and the importance of building supportive environments [29]. A key finding in our review is that studies in low- and middle-income countries (LMICs), reflecting impact from local conditions, reported infrastructural and technical limitations to implement mHealth ranging from low network capacity, inadequate distribution of signal strength, and low access to mobile phones to specific technical barriers, such as delay in database updating. These observations add relevance to the suggestion that cooperation between government organizations, academia, and industry is necessary to provide sufficient infrastructure support for mHealth use in LMICs [30].

Another main result of our review is that only one article [24], reporting a 6-month intervention involving healthy Swedish children aged 4.5 years via a smartphone application that provided educational information, addressed primary outcomes. The authors found no difference between the intervention and control group concerning FMI measurements. This can be resulted from several factors; one of them considered to be inclusion of all kids regardless of baseline FMI, i.e. also children with normal weight and body fat. The mHealth interventions period was limited and these interventions did not fundamentally change the anthropometric measurements. However lifestyle were promoted changes that may ultimately result in healthier body composition. For providing mHealth interventions that impact anthropometric outcomes among children and adolescents, it is suggested that the duration of interventions measuring BMI levels extend longer than 20 weeks [31]. Moreover, it has been argued that a high quality economic evaluations of public health intervention is needed which can estimate costs and effects which looks more responsive to the needs of the decision makers using them [32–34]. Contrary to our expectations, none of the included studies assessed the economics aspect of the investigated health application in a clear way, but the authors of one article indicated an economic evaluation of the program will be performed in the future [23].

The main mechanism by which mobile phone technologies support at risk of nutrition-related disorders is by raising patient involvement and increasing self-management abilities [35, 36]. The most frequent barrier towards combating pediatric nutritional problems that was expressed by study practitioners was lack of parent involvement [37]. But in contrast to adults who can accept the responsibility of monitoring and adjusting their own treatment, the liability of nutritional interventions in preschoolers is by their parents [38, 39]. Therefore, guidance in parenting methods and behavioral management procedures should be considered as key areas in mHealth interventions aimed at preventing or treating children’s nutrition-related problems [37, 40].

Although the insights gained from our study provided positive signals concerning the acceptance of mobile phone based nutritional interventions addressing preschoolers, obstacles such as lack of time, cost, support staff, right referral resources, concern about parental responses, and lack of knowledge, skills and training in the area were reported. For instance, sever variations in compatibility between manufacturers affecting the use of a barcode scanner, have been reported. As the solution, the authors suggested use of the iPhone Operating System (iOS) platform, known seldom to be associated with compatibility issues. It can thus be recommended that logistic support, staff time, consumer preference, and cost factors are investigated before starting mHealth programs. Thoroughly understanding stakeholders’ utilization of mobile phones can be helpful in recognizing key indicators identified with feasibility and ideal usage of such innovation to avert or potentially diminish the rate of wellbeing related issues [41]. Several studies have indicated generally positive attitudes toward telecommunication and mHealth strategies [42, 43]. A qualitative study among health care providers that surveyed attitudes towards mHealth reported that potential advantages of incorporating mHealth technology also include the lessening of literacy obstructions, writing mistakes and redundancy, and an expanded feeling of privacy for the clients, even though significant concerns expressed by providers included increased network connectivity needs and liability for the devices [44].

In our review only one of the studies has used an intervention that is relied on CBSB. mHealth tools have the potential to become involved with users in real time and in highly tailored ways to encourage healthy habits, based on collection of personal data, gathered feedbacks from the user and activity levels [45, 46]. There are some concerns about incorporating evidence-based content and theory-based strategies alongside promoting behavioral changes. Several investigators have suggested for using of models to inform the design of Behavioral Intervention Technologies (BITs) as a key strategy [47–51]. For example Fogg in his model introduced behavior as a product of three factors: motivation, ability, and triggers which must occur at the same moment, else the behavior will not happen [49]. But in another study by David C Mohr et al. these models have been said offer little information on how to design and implement BITs. So an expansive hybrid framework that consolidates behavioral standards with technological aspects has been suggested by them. This framework can be helpful to connect the fields of behavioral science and technology [52].

## Limitations

Limitations in this systematic review included a risk of review bias. Important findings may have been overlooked from excluded studies not published in English or that involved mobile application interventions for children and adolescents out of our age criteria. The small number of available studies, especially randomized controlled trials, limits this review. The use of applications for nutrition education is relatively new, and there is much research to be done.

## Conclusion

A key finding in our review is that studies in LMICs reported important infrastructural and technical limitations to implement mHealth ranging from low network capacity, inadequate distribution of signal strength, and low access to mobile phones to specific technical barriers, such as delay in database updating. Also, only one study was identified evaluating primary anthropometric outcomes, indicating a need for more controlled studies of these primary endpoints. The review findings put relevance to the suggestion that cooperation between government organizations, academia, and industry is necessary to provide sufficient infrastructure support for mHealth use in LMICs. They also point to a need for enhanced strategies to motivate intervention participation by use of means such as newspaper and social media advertisements, health workers advice, and motivational gifts.

While several review studies have examined the role of mobile technology in nutritional interventions to promote proper diet and nutrition or to lose weight in adults [10, 16, 53] and adolescents [19], to the best of our knowledge, no reviews have focused on the role of mobile for addressing malnutrition (both under-nutrition and over-nutrition) issues for the sensitive age group of preschoolers which is critical in their growth and development. Furthermore, one featured points of our study is an almost comprehensive evaluation of the interventional articles in an extensive domain including users’ perceptions, technical and usability, usage and adoption factors and Efficacy of the intervention, which can provide significant opportunities for future research by concerning wide aspects.

## Abbreviations

AWWs: Anganwadi Workers
BITs: Behavioral Intervention Technologies
BMI: Body Mass Index
CBSB: Cognitive Behavioral Skills Building
E-Health: Electronic Health
FMI: Fat Mass Index
ICT: Information and Communication Technologies
iOS: iPhone Operating System
IYCF: Infant and Young Child Feeding
LMICs: low- and middle-income countries
M-Health: Mobile Health
MINSTOP: Mobile-based Intervention Intended to Stop Obesity in Preschoolers
MVPA: Moderate-to-Vigorous Physical Activity
PHM: Public Health Midwives
PRISMA: Preferred Reporting Items for Systematic reviews and Meta-Analyses
RCT: Randomized Controlled Trial
SMS: Short Message Service
WHO: World Health Organization
WIC: Women, Infants, and Children
